# Supplementary material for: Molecular pathway analysis towards understanding tissue vulnerability in spinocerebellar ataxia type 1
Source: eLife. 2018 Dec 3;7:e39981. doi: 10.7554/eLife.39981 (PMC6292693; doi:10.7554/eLife.39981)
Supplement: Transparent reporting form [file elife-39981-transrepform.docx]

***eLife’s* transparent reporting form**

We encourage authors to provide detailed information *within their submission* to facilitate the interpretation and replication of experiments. Authors can upload supporting documentation to indicate the use of appropriate reporting guidelines for health-related research (see [EQUATOR Network](http://www.equator-network.org/%20)), life science research (see the [BioSharing Information Resource](https://biosharing.org/)), or the [ARRIVE guidelines](http://www.plosbiology.org/article/info:doi/10.1371/journal.pbio.1000412) for reporting work involving animal research. Where applicable, authors should refer to any relevant reporting standards documents in this form.

If you have any questions, please consult our Journal Policies and/or contact us: [editorial@elifesciences.org](mailto:editorial@elifesciences.org).

**Sample-size estimation**

- You should state whether an appropriate sample size was computed when the study was being designed
- You should state the statistical method of sample size computation and any required assumptions
- If no explicit power analysis was used, you should describe how you decided what sample (replicate) size (number) to use

Please outline where this information can be found within the submission (e.g., sections or figure legends), or explain why this information doesn’t apply to your submission:

For RNA-sequencing, it is recommended to use at minimum 2-3 biological replicates per group. This number of biological replicates is consistently used in the published literature. In the present study, we utilized 3 biological replicates per group, and found that gene expression changes were robust (FDR p-value < 0.05), and were verified by RT-qPCR. We deemed the rationale of using 3 biological replicates for RNA-sequencing as common knowledge, and is thus not reported in the manuscript.

No pre-determination of sample size or sample-size calculation was performed prior to experimentation. In our previous experience with similar experiments (Ju et al., Journal of Neuroscience 2013; Todd et al., eLife 2015), we have determined that a small sample size is sufficient for our analysis, therefore, we used similarly size groups.

**Replicates**

- You should report how often each experiment was performed
- You should include a definition of biological versus technical replication
- The data obtained should be provided and sufficient information should be provided to indicate the number of independent biological and/or technical replicates
- If you encountered any outliers, you should describe how these were handled
- Criteria for exclusion/inclusion of data should be clearly stated
- High-throughput sequence data should be uploaded before submission, with a private link for reviewers provided (these are available from both GEO and ArrayExpress)

Please outline where this information can be found within the submission (e.g., sections or figure legends), or explain why this information doesn’t apply to your submission:

Information regarding replicates is clearly reported in each corresponding figure legend, methods section, and results section. No data (i.e. outliers) were excluded in this study.

The GEO accession number for RNA-seq data generated in this manuscript is GSE122099. Our GEO accession number is currently private. A link for reviewers is provided in the Response to Reviewer's document, and is listed below. To review GEO accession GSE122099, click the following link and enter token olglqsiwjbkrhit into the box:

[https://na01.safelinks.protection.outlook.com/?url=https%3A%2F%2Fwww.ncbi.nlm.nih.gov%2Fgeo%2Fquery%2Facc.cgi%3Facc%3DGSE122099&amp;data=02%7C01%7Cterri.driessen%40yale.edu%7Cab5f1c8748154cd1028708d64406126f%7Cdd8cbebb21394df8b4114e3e87abeb5c%7C0%7C0%7C636771190224299882&amp;sdata=wLEQ1vVCQcOvU5L4EdvgwyaGr8XnfSrTSyzX%2FNBBeno%3D&amp;reserved=0](https://na01.safelinks.protection.outlook.com/?url=https%3A%2F%2Fwww.ncbi.nlm.nih.gov%2Fgeo%2Fquery%2Facc.cgi%3Facc%3DGSE122099&data=02%7C01%7Cterri.driessen%40yale.edu%7C22ddafaccf8e49b2890508d644069af5%7Cdd8cbebb21394df8b4114e3e87abeb5c%7C0%7C0%7C636771192482415296&sdata=SKyLVUBNS88Z5uqhf6UlptF0fwteyavZ56lKU4CKl%2B8%3D&reserved=0)

**Statistical reporting**

- Statistical analysis methods should be described and justified
- Raw data should be presented in figures whenever informative to do so (typically when N per group is less than 10)
- For each experiment, you should identify the statistical tests used, exact values of N, definitions of center, methods of multiple test correction, and dispersion and precision measures (e.g., mean, median, SD, SEM, confidence intervals; and, for the major substantive results, a measure of effect size (e.g., Pearson's r, Cohen's d)
- Report exact p-values wherever possible alongside the summary statistics and 95% confidence intervals. These should be reported for all key questions and not only when the p-value is less than 0.05.

Please outline where this information can be found within the submission (e.g., sections or figure legends), or explain why this information doesn’t apply to your submission:

Information pertaining to statistical testing, sample size, and p-values for each analysis is included within the figure legends, methods section, and results section.

(For large datasets, or papers with a very large number of statistical tests, you may upload a single table file with tests, Ns, etc., with reference to sections in the manuscript.)

**Group allocation**

- Indicate how samples were allocated into experimental groups (in the case of clinical studies, please specify allocation to treatment method); if randomization was used, please also state if restricted randomization was applied
- Indicate if masking was used during group allocation, data collection and/or data analysis

Please outline where this information can be found within the submission (e.g., sections or figure legends), or explain why this information doesn’t apply to your submission:

Inclusion of animals in a specific experimental group was determined by the animal genotype. Because there was no pharmacological manipulation of any of the experimental groups tested, animals were not randomized. For immunofluorescence measurements, the observer was blinded to the animal genotype, and cell counts and fluorescence intensity were calculated in ImageJ.

**Additional data files (“source data”)**

- We encourage you to upload relevant additional data files, such as numerical data that are represented as a graph in a figure, or as a summary table
- Where provided, these should be in the most useful format, and they can be uploaded as “Source data” files linked to a main figure or table
- Include model definition files including the full list of parameters used
- Include code used for data analysis (e.g., R, MatLab)
- Avoid stating that data files are “available upon request”

Please indicate the figures or tables for which source data files have been provided:

Source data for Figures 2-6 have been provided (Figure 2 - source data 1-5; Figure 3 - source data 1-5; Figure 4 - source data 1-4; Figure 5 - source data 1-4; Figure 6 - source data 1, 2).

The names of software and version number are listed in the Key Resource Table and text. Parameters are listed in the Materials and Methods section.
